# Internet-of-Things Based Hardware-in-the-Loop Framework for Model-Predictive-Control of Smart Building Ventilation

**DOI:** 10.3390/s22207978

**Published:** 2022-10-19

**Authors:** Abdelhak Kharbouch, Anass Berouine, Hamza Elkhoukhi, Soukayna Berrabah, Mohamed Bakhouya, Driss El Ouadghiri, Jaafar Gaber

**Affiliations:** 1 LERMA Lab, College of Engineering, The International University of Rabat, Technopolis Rabat-Shore Rocade Rabat-Salé, Sala El Jadida 11100, Morocco; 2 I&A Laboratory, Faculty of Science, Moulay Ismail University of Meknès, B.P. 11201 Zitoune, Meknès 50070, Morocco; 3 ENSIAS, Mohamed V University, Rabat 10713, Morocco; 4 Univ. Bourgogne Franche-Comte, UTBM, FEMTO-ST UMR CNRS 6174, 25000 Belfort, France

**Keywords:** Internet of Things, model predictive control, hardware in the loop, machine learning, energy efficiency, smart buildings

## Abstract

In this work, a Hardware-In-the-Loop (HIL) framework is introduced for the implementation and the assessment of predictive control approaches in smart buildings. The framework combines recent Internet of Things (IoT) and big data platforms together with machine-learning algorithms and MATLAB-based Model Predictive Control (MPC) programs in order to enable HIL simulations. As a case study, the MPC algorithm was deployed for control of a standalone ventilation system (VS). The objective is to maintain the indoor Carbon Dioxide (CO_2_) concentration at the standard comfort range while enhancing energy efficiency in the building. The proposed framework has been tested and deployed in a real-case scenario of the EEBLab test site. The MPC controller has been implemented on MATLAB/Simulink and deployed in a Raspberry Pi (RPi) hardware. Contextual data are collected using the deployed IoT/big data platform and injected into the MPC and LSTM machine learning models. Occupants’ numbers were first forecasted and then sent to the MPC to predict the optimal ventilation flow rates. The performance of the MPC control over the HIL framework has been assessed and compared to an ON/OFF strategy. Results show the usefulness of the proposed approach and its effectiveness in reducing energy consumption by approximately 16%, while maintaining good indoor air quality.

## 1. Introduction

Heating, ventilation, and air-conditioning (HVAC) systems are considered among the main building’s energy consumers. They account for approximately 50% of the global energy usage in buildings and 36% of all energy-related CO_2_ emissions worldwide [1,2]. Therefore, HVAC systems need to be efficiently designed and controlled, in reference to international standards, to ensure optimal trade-off between the occupants’ comfort and energy efficiency in buildings [3,4]. On the other hand, to assess the energy performance in the design of HVAC management services in buildings, four main comfort metrics need to be considered, which are the visual comfort, acoustic comfort, thermal comfort, and the Indoor Air Quality (IAQ) [5]. This latter has been identified as one of the most important metrics influencing the indoor environmental comfort of the occupants as well as one of the main sources of energy consumption in buildings [4,6], which depends mainly on standalone ventilation management systems.

On the other hand, the indoor concentration of CO_2_ is considered among the most important parameters for developing efficient control strategies of VSs [7]. The aim is to minimize their electrical energy consumption, while providing good IAQ to the occupants. The objective is to keep the CO_2_ concentrations within the comfort range by providing the required fresh air from outside to the inside of the building using optimal ventilation flow rates. Basically, the majority of conventional building’s VSs are operated by simple rules-based controllers (e.g., intuitive ON/OFF controllers or simple PID controllers). Most of them are based on predefined operating parameters, which use normal ventilation rates, to provide the amount of outside air demanded by the building. However, their control mechanism is still inefficient regarding the performance decrease in frequent system context changes as well as dealing with the time-delay [8]. Typically, the ventilators acts automatically on behalf of many buildings’ context-awareness parameters, such as the indoor temperature (based on the envelope characteristics), control modes of the VSs, and occupants’ presence [9]. This can affect the energy operation flexibility, indoor environmental comfort, and occupants’ productivity due to uncontrollable ventilation rates, resulting in wasted energy [10].

Recent studies highlighted that weather conditions and occupants’ behavior are the most important information that can help to improve a building’s services (e.g., VSs) [7]. Occupancy detection systems in buildings are mostly involved in extracting meaningful occupancy information, which could be used for setting up different control strategies. Different occupancy parameters can be collected from the building’s environment including occupants’ presence, number, activity, identity, location, and tracks. All of these metrics can be integrated in Building Energy Management Systems (BEMS) as a primary input for controlling active/passive systems, such as HVAC, standalone ventilation, and lighting [11,12,13,14]. Most recent research work investigated the development of intelligent methods by integrating machine learning, deep learning, and reinforcement learning [15,16]. In addition, advanced techniques from automation, system modeling and optimization, Internet of Things (IoT) for real-time systems monitoring, data processing and context-aware computing techniques could be combined for the development of BEMS [17]. 

As is commonly known, new innovative designs of equipment and system components need to be tested while going through extensive essays [18]. The aim is to validate and properly ensure their reliability before deploying them in real-sitting scenarios. The tests can either be run in a laboratory (small scale), using only pure simulations, or by combining both ways, resulting in HIL simulations [19]. Unlike conventional simulations, concrete testing in laboratories may be seen as the most accurate and is a sure indicator of performance. However, it has some limitations. First, it can generate high costs and is subject to many constraints. For instance, the number of tests that can be run over a period of time and under the same conditions are very limited. Hence, comparing different control approaches or different products providing the same function becomes challenging. On the other hand, numerical simulation is another used method among engineers and researchers to properly evaluate the performances of control methods of many applications, which can be deployed in buildings or other sectors. Numerical models could capture the dynamic of the equipment and the building while considering real weather conditions (if available), the combined internal loads (gains, lighting, occupancy, etc.), and other stimuli. Furthermore, once the numerical model is mature enough, it can be used repetitively to evaluate equipment’s control at lower costs. For this, the model should prove its fidelity and accuracy in mimicking the real system’s behavior and the related building. As can be noticed, a variety of validated models and toolkits are available for a variety of domains using different simulation tools [20]. It is worth mentioning that, during the recent decades, co-simulation capabilities expanded the modeling scope further to other domain systems at a very precise resolution [21]. However, some problems cannot be tackled easily through simulations, especially if numerical models cannot capture all necessary details [22].

In parallel, recent advances in IoT and big data technologies allow for real time data monitoring and processing, while enabling predictive analytics and advanced systems’ control. In fact, IoT is considered the most important emerging technology, allowing for the development of advanced and smart connected solutions varying from eHealth, industry and transportation to energy management and smart control [23,24,25,26,27,28,29,30,31]. Any system or device having the capability to connect to a network and communicate over the Internet is considered a thing in an IoT infrastructure [32]. This latter provides required tools to manage, control, monitor, visualize and process the things’ data (e.g., embedded devices, smartphones, smart actuator, sensors). In parallel to this progress, the integration of smart energy grids with IoT and big data techniques has recently emerged into what is named the Internet of Energy or Energy Internet [19,20]. In fact, with the emergence of smart power meters and smart electrical appliances, it is now possible for users to closely monitor energy consumption while having the ability to plan and manage their consumption. The IoT infrastructure makes it possible to capture and analyze sensor data in real time, allowing consumers to interact with data and decision making [33].

The main aim behind the framework proposed in this paper is to fill the gap between simulations and real case experimental validation of control approaches and mechanisms. The framework could be used not only to control buildings systems but also for other use cases in which the experimental validation of a developed control model is needed. A flexible architecture of the platform has been introduced and its components are detailed to provide an easy to implement solution for similar applications. The work presented in this paper focuses on the integration of IoT/big data techniques with simulation tools in order to enable HILS. The aim is to join both field testing and numerical modeling by combining hardware and software to form HILS frameworks. These latter make it easy to assess multiple tests under the same conditions and, eventually, to accommodate for dangerous operations. As a case study, to show the usefulness of the HILS framework, a Model Predictive Control approach (MPC) was deployed on standalone VS. The framework integrates recent IoT and big data platforms together with machine-learning algorithms and MATLAB-based MPC model.

In summary, the objective of the work is twofold, first to show the usefulness of the proposed IoT based HIL framework together with the integrated machine learning model and smart control technique of MPC, and second, to study the performance of the MPC model combined with forecasted occupancy number and real-time test site’s contextual data. The goal of the experimentation is to maintain the indoor CO_2_ concentration at the standard comfort range while enhancing the energy efficiency. Setting up a field operational testing predictive control techniques is a very challenging and time consuming task. This work could leverage the gap between simulations and real application of predictive control in smart buildings.

The remainder of this paper is structured as follows. Section 2 presents recent work related to advanced strategies for smart control and IoT-HIL based approaches. In Section 3 and Section 4, the description of the used materials as well as the architectures of the proposed control strategies and the IoT-HIL platform will be presented. In Section 5, results are presented to demonstrate the accuracy of the proposed models as well as the developed framework. Conclusions and perspectives are presented in Section 6.

## 2. Related Work

Recent research work showed that reducing energy consumption in buildings, especially those related to HVAC systems, can be attained through the usage of advanced control strategies. In this regard, two main approaches of rule-based control algorithms have recently emerged in the field of advanced HVAC control: Learning based approaches (e.g., fuzzy logic, Artificial Neural Networks (ANNs), fuzzy and adaptive fuzzy neural networks and genetic algorithms) and MPC [34]. Among these control algorithms, MPC has been introduced as one of the most powerful control techniques used to manage complex processes, such as in HVAC systems [35] studies. This control technique can handle nonlinear processes and their dynamics according to different objectives functions, such as those related to indoor air quality and thermal comfort improvement [36,37].

On the other hand, one of the most efficient ways of conducting field operational testing appears to be the HIL simulations seeing its various advantages (low cost, accurate results, etc.) [38]. This new concept is becoming widely used in developing and testing complex real-time embedded systems [39]. This is mainly done by adding, through mathematical representations (also referred to as “plant simulation”), the complexity of the plant to be controlled into the test bed [40]. To perform HIL simulations, electrical emulation of sensors and actuators is used to interface between the “plant simulation” and the “system under test”. In fact, the plant simulation controls the value of the emulated sensor, which is then read by the embedded system under test. In HIL simulations for system synthesis, major physical equipment and their associated controllers are integrated with simulated devices or building spaces to investigate behaviors under realistic dynamic conditions.

In the last decade, researchers focused their interest on the HIL approach and used it not only in automotive and spatial systems but also in buildings’ equipment testing and control. For instance, Missaoui et al. [41] proposed new BEMS strategies to support demand side management and to validate them using a Power-Hardware-in-the Loop (PHIL) test bench. However, the proposed solution can be used to validate control algorithms in a reasonable time. Schneider et al. in [42] focused their work on investigating the interaction of a real circulating pump with the hydronic network of a virtual building energy and control system. The presented model, using Modelica for building simulation, is used to bridge the gap between the design and commissioning stage of a control algorithm for HVAC components. The used model is a single-family dwelling with limited complexity. The comparison between simulation results and measured data proved the accuracy of the model with a mean relative error less than 4%. De la Cruz et al. in [43] presented, in their paper, the implementation of an HIL real time simulation test bunch for Air-to-Water-Heat-Pumps (AWHP). This will allow HVAC manufacturers to optimize the control of their systems and to improve their efficiency. A real AWHP was tested under real climate conditions, as for the thermal loads, they were calculated through the connection of the AWHP and a virtual building, simulated using Modelica software, via HIL real time simulation. Seifried et al. in [44] proposed a new model, based on the interconnection of a prominent building automation protocol, namely BACnet, and the PowerDEVS simulator to facilitate HIL testability of new and existing building automation system components. Huang et al. in [22] presented an agent-based framework for HIL simulations, which could either be used for investigating the controller performance or HIL for system synthesis. In other words, it is possible to involve controllers as well as other major equipment in the test to ensure that their dynamic behavior is being correctly captured. Zahari et al. [45] developed a control algorithm to bring the HIBORO helicopter prototype into equilibrium. The developed algorithm is a combination of the MPC and the black box nonlinear autoregressive model. Using the Xpc Target rapid prototype under Simulink, HIL simulations have been run for different set points to evaluate the performances of the proposed model. This latter contains inertial measurement unit sensor software, the MPC, and C/T blocks for capturing and generating Pulse Width Modulation (PWM) signals. The controller proved its efficiency in terms of stabilizing the prototype under all disturbances.

Samano-Ortega et al. [46] developed a platform for the validation of photovoltaics (PVs) system controllers using IoT and HIL concept. The platform englobes five main parts: (i) a control emulator based on HIL, producing the behavior of PVs’ arrays, a converter, and Alternating-Current (AC) loads, (ii) Cloud database, (iii) smart sensors for load monitoring, (iv) residential PVs (RPVs) connected to the Internet, and (v) a mobile application for tracking and monitoring. The main principle is that measured voltage and current of the AC loads (using smart sensors) and the production of RPVs are downloaded to the HIL, which reproduces the behavior of the PVs and loads in real-time. The platform proved its efficiency in emulating the behavior of the installed PVs with a mean relative error of 0.42% and the AC load with a mean absolute error of 10 mA. Conti et al. [47] showed the relevance of the dynamic coupling between an air-source heat pump and a building apartment, located in Pisa (Italy), in winter in terms of energy performances under three different operational modes. The adopted HIL extensive experimental campaign proved its potential in properly estimating the energy consumption as well as developing advanced operational strategies. Frison et al. [48] developed a simple low cost MPC controller, which has been evaluated using HIL experiments, for assessing, under realistic conditions, the energy performances of a heat pump system.

Furthermore, due to the large availability of smart low-cost embedded devices (e.g., Arduinos, Raspberry pi, NVidia Nano, actuators, and distributed sensors), and data streaming processing tools, such as Storm/SAMOA and Kaa applications [49], and generally, the advances of information and communication of IoT technologies [50], the implementation of optimal control strategies for improving the energy efficiency as well as indoor air quality and thermal comfort is becoming immediate and more viable [51]. Their application has been widely studied for the development and deployment of intelligent context-aware services and applications, such as occupancy prediction [52], healthcare [31], transportation and logistics [53], smart grids [54,55], and smart homes [56]. For example, Huchuk et al. [16] evaluated numerous classification machine learning algorithms and models for predicting occupants’ presence in smart buildings using thermal data. Further, Zhang et al. [57] presented a literature review about the integration of machine learning for predicting occupancy patterns to improve indoor air quality, while optimizing energy use. In addition, online machine learning techniques (e.g., vertical Hoeffding tree and self-adjusting memory for KNN) can be included for predicting occupants’ number and presence using environmental data, such as CO_2_ temperature and humidity [58,59]. IoT and HIL concepts could provide an integrated solution to cover the important aspects of BEMS by enabling the collection, monitoring, and processing of stream data together with machine-learning techniques. These latter are, for instance, used to compute accurate forecasts, which are required for the MPC to compute accurate predictions, i.e., forecast optimal actions for real-time control of a building’s services. 

In this work, a case study that focuses on a standalone VS, is worked out to assess the usefulness and effectiveness of the proposed HIL framework. In fact, data that has been collected from a set of sensors, such as temperature, motion, and CO_2_ concentration, is used to predict occupancy patterns [30]. These latter are then fed to the MPC to control indoor CO_2_ dynamics by forecasting the optimal ventilation rates.

## 3. Materials and Methods

In this section, an HIL experiment of a closed-loop VS driven by the MPC is performed. The MPC ventilation controller model, which has been previously designed, developed, and validated using simulations [28,29], has been physically deployed to the Raspberry Pi (RPi) located at the EEBLab test site. The RPi-in-the-loop experiment has been run under realistic conditions to dynamically actuate the fans of the VS and to assess the controller’s performance in terms of energy efficiency and indoor CO_2_ improvement. In fact, the deployed VS is made of two standalone controlled fans, which are respectively responsible for bringing the fresh outdoor air to the indoor and draining the CO_2_ out of the building. More precisely, these two fans are installed in both side walls and operate instantaneously under the same control signals. The VS can provide a maximum airflow rate of 440 m^3^/h, which is equivalent to a rated speed of 3800 rpm and is powered by a photovoltaic solar system. The occupancy information was used as a disturbance as well as a forecast input for the MPC.

### 3.1. Description of the Case Study Building: EEBLab

**Figure 1:**
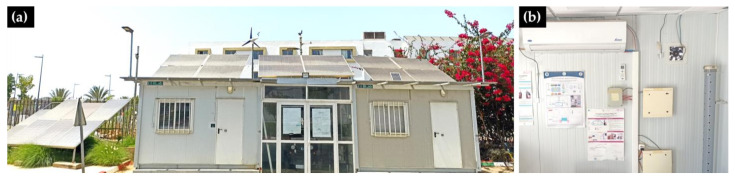
(**a**) Energy Efficient Building Lab (EEBLab) test site; (**b**) Interior side wall of EEBLab with ventilation fan and a set of sensors and other equipment.

The considered specimen, named Energy Efficient Building Laboratory (EEBLab), is a rectangular cavity, which is part of a set of two identical prefabricated structures (Figure 1), located at the International University of Rabat. Each test bed is 12 m^2^ of occupied surface and 30 m^3^ of volume. Additionally, each prefabricated has one single glazed window on its south façade. The laboratory has been made essentially for implementing and testing different scenarios related to eHealth, Energy efficiency, ICT, and renewable energies integration and control. The main aim is to investigate the integration of recent IoT, big Data technologies, and advance real-time machine learning algorithms for developing context-aware services and applications.

### 3.2. The HOLSYS Internet of Things Platform Setup

**Figure 2:**
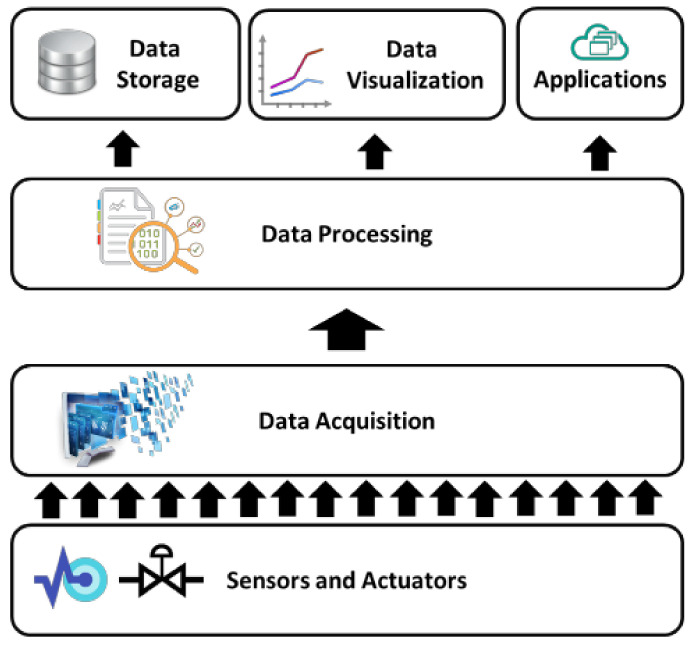
General architecture of an Internet of Things platform.

The HOLSYS platform has been in development and passed from many stages namely, the use of Kaa project IoT platform and the upgrade to the ThingsBoard open source IoT platform. It allows configuring, supervising, and acquiring connected sensing and actuating nodes. Its aim is to allow the development and deployment of IoT based scenarios related to smart energy efficient buildings as well as eHealth and Smart Mobility. The HOLSYS platform follows the general architecture presented in Figure 2. The four layers define the different general sections/aspects of an IoT platform, namely, Sensing/actuating, Data Acquisition, Processing, and Visualization.

#### 3.2.1. Sensing and Actuation Layer

Sensors and actuators represent all embedded sensors and actuators together with control units considered as one device and presented to the platform as an IoT node. This latter is capable of receiving and sending stream data while ensuring the execution of all control strategies sent by the platform. The communication between deployed nodes and the HOLSYS platform may pass through wired or wireless protocols. In this paper, only MQTT, REQUEST and REST have been used as the EEBLab is accessible in the campus network either wirelessly or through an ethernet connection.

The deployed IoT devices (nodes) are built using low-cost microcontrollers (e.g., NodeMCU, Arduino, STM32S) and presented to the platform using Raspberry Pi 3 and 4 B+ (RPi). A set of nodes can be connected in serial mode (USB), Serial Peripheral Interface (SPI), Inter-Integrated Circuit (I2C) or Bluetooth to the RPi gateway where NodeRed controls the inputs and the outputs of the set. NodeRed is a flow-based platform, developed originally by IBM, for facilitating the process of wiring hardware devices together with Application Programming Interfaces (APIs) and online services as part of the Internet of Things. A NodeRed flow is a set of connected logical NodeRed nodes linked together to form a processing logic with inputs and outputs. The resulting logic flow ensures gathering input data from wired or wireless IoT nodes, pre-processing and aggregating them to finally output structured data into local or remote storage systems.

**Figure 3:**
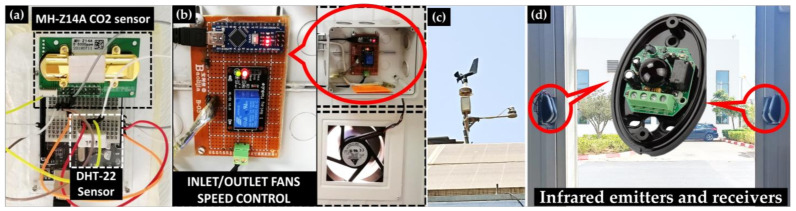
The deployed IoT devices; (**a**) Indoor CO_2_, temperature and humidity node; (**b**) Inlet and outlet ventilator speed control node; (**c**) Weather station node for outdoor air quality; (**d**) Occupants’ number node.

Figure 3 presents the deployed sensors and actuators used in this study. (a) represents the Indoor air quality node connected to an RPi via a USB cable. It gathers the indoor CO_2_ concentration in Part Per Million (PPM) using an MH-Z14A sensor with an accuracy of ±50 PPM +3% reading value; (b) shows the control node of both inlet and outlet fans used to ventilate the EEBLab. The 12 V and 440 m^3^/h fans are controlled with PWM generated from an Arduino nano. Speed can be controlled from 0, for OFF mode, to 255 PWM for max speed (ON mode). However, a relay has been added to completely turn OFF the fans if 0 PWM has been triggered to save energy; (c) presents the deployed RPi based weather station with wind speed and direction, solar irradiance, ambient temperature and relative humidity sensors for outdoor environmental data. Used sensors are, respectively, an analog anemometer and magnetic direction sensors, SR20 pyranometer and DHT22 together with DS18B20; (d) depicts gate door motion sensors to determine the true occupants’ number inside the EEBLab. They are based on infrared emitters and receivers, which are aligned together at the door entrance, to detect the exact occupants’ number.

#### 3.2.2. Data Acquisition Layer

**Figure 4:**
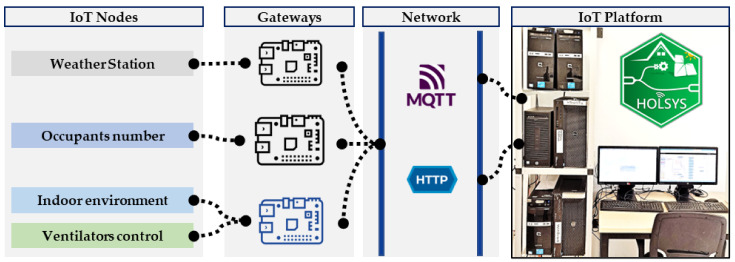
Data transfer from IoT nodes to the HOLSYS platform via RPi gateways over MQTT and HTTP.

Data collected at the first layer (Sensing and Actuation), using the deployed sensors, are sent via HTTP requests and MQTT by the RPi gateways to the HOLSYS platform, which is deployed in the remote server, as depicted in Figure 4. The HOLSYS platform is based on the open source Thingsboard IoT platform in its community edition. Services and packages together with all connectors enabling the acquisition of all the deployed IoT devices are installed and configured in the cluster composed of one performant master and three slaves. They are HP computers with Intel core i3 and i5 with 4 Gbytes of RAM and 500 Gbytes of storage each.

**Figure 5:**
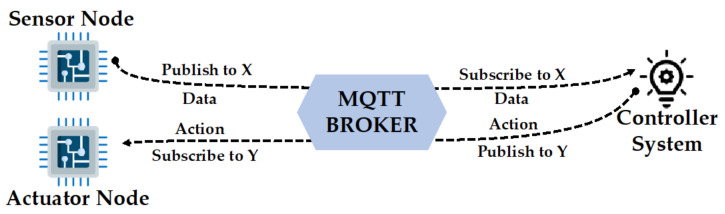
MQTT stream data flow from/to sensor/actuator/controller.

The HOLSYS acquisition layer is a set of RPis representing the deployed nodes to the platform. A RPi is a tiny credit-card sized computer using the Raspbian operating system, a free version based on Debian and optimized for its limited power. The three gateways that have been deployed are a 4 Gbyte RAM RPi 4 B+ and two 1 Gbyte RAM RPi 3 B+. NodeRed is used in these RPis to acquire data from the serial connected nodes to pre-process and store them locally in files for a backup. Each node is represented to the platform by a unique token and identifier, which is used to secure the communication and to identify the streamed data. HTTP requests have been used as a backup transfer protocol in case the MQTT broker becomes unfunctional. However, the HTTP protocol is not suitable for IoT architectures as it is more energy consuming and has a bigger data packet size. On the other hand, MQTT has been designed to provide a lightweight messaging technique enabling small packet size for faster transfer. The MQTT is an IoT data transfer protocol having publish/subscribe architecture. It is based on a broker to which all clients, either subscribers, publishers, or both at the same time, should be connected to (see Figure 5).

The installed broker is the central communication point where data is exchanged between clients based on a topic. This latter is a category in which a given client has published data. From the other side, the subscriber will get the data from the given topic.

#### 3.2.3. Data Processing Layer

**Figure 6:**
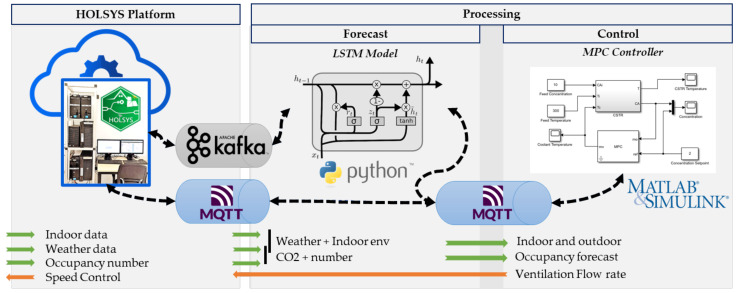
General architecture platform for controlling ventilation system based on occupancy forecast and CO_2_ measurement.

The processing of data can be performed in real-time or batch manners in the HOLSYS platform servers or by third-party applications deployed elsewhere. The local processing can be a simple aggregation of each received tuple of data or a complex processing using the rule-based engine. It is a customizable and configurable system for complex event processing. It allows for filtering, enriching, and transforming incoming data and triggering various actions, for example, notifications or communication with external systems. In the current study, a rule chain has been implemented to save received data to the HOLSYS database (NoSQL Cassandra) after applying filters on them according to each data source (e.g., Temperatures, Humidity and CO_2_ ranges; null values, missing data) and forward, via MQTT and Kafka, the filtered data to external sources for other applications. This latter plays a key role for integrating machine learning applications. For instance, the occupancy forecast, an important input to the MPC algorithm, is performed as an external application while using the filtered data coming from the platform. In fact, Apache Kafka, a high-performance real-time data streaming technology capable of handling large amounts of events, is used as a pipeline to transmit stream data between the platform and other applications (e.g., machine learning algorithms). This part shall be detailed in the next section. Furthermore, MQTT has been used to send data to the consumers in the MATLAB MPC control as it is supported by default as a communication method implemented by MATLAB. Figure 6 depicts the communication between the platform and the processing layer. Kafka and MQTT are the main tools used to allow the processing layer to receive data for occupancy forecasts and MPC control. For instance, the forecast model receives the indoor CO_2_ concentration and the real occupancy number, respectively over MQTT and Kafka, to forecast 10 steps ahead. Real-time forecasted values are fed as an input to the MPC controller in order to predict the required control actions, which are sent back to the HOLSYS platform for execution.

#### 3.2.4. Data Storage, Visualization, and Applications Layer

This layer presents all external services that can be connected to the platform by means of supported data transfer protocols and technologies. Mainly, Kafka, MQTT, and HTTP requests are used to allow external third-party applications to connect, produce data, and consume available resources of the HOLSYS platform. In addition to the local NoSQL Cassandra database, which is deployed by default for storing data at the platform level, Mongodb is also used to store backup data for batch processing, serving other applications, such as training the forecast model of occupancy. The main reason behind using Mongodb is its architecture based on storing data as JSON format but with a special syntax called BSON. Each set of data is stored as a document into a collection while its content can be unstructured and different from other documents, resulting in the NoSQL principle, which does not need a tabular and relational concept. Furthermore, the collected data is broadcasted on MQTT using adequate topics for all other applications, mainly those requiring shared resources, such as weather data. As for data visualization, Grafana tool is being integrated with Cassandra and the Mongodb database for real-time visualization of data streams.

### 3.3. Data Measurement/Preparation for Occupancy Prediction

Occupancy information, as stated above, is one of the most important inputs to context-driven control approaches for efficient control of building equipment. Several studies show the effectiveness of integrating occupancy in MPC for HVAC, ventilation and lighting systems control [60,61]. In order to collect accurate occupancy prediction, different techniques can be used, including PIR sensors, cameras, RFID, Wi-Fi, Bluetooth-low-energy (BLE), and environmental data (e.g., CO_2_, temperature, and humidity) [62,63,64].

**Figure 7:**
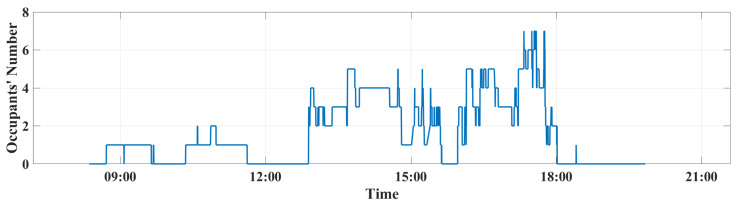
Occupants’ numbers over the day in EEBLab.

In this work a data set containing almost 28,000 instances for one day from 8:30 until 19:00 has been used. Data are collected from EEBLab using the occupancy number node and stored into a Mongodb data (see Figure 7). The occupancy profile varied between one and seven occupants during the day. 

Deep learning-based occupancy forecasting techniques have been investigated [65]. The first two recurrent neural network (RNN) based methods, long short-term memory (LSTM) and gated recurrent unit (GRU), have been evaluated and compared in terms of accuracy and root mean square error. These algorithms are classified as extensions of RNN by integrating internal gates which help in deciding whether to keep or throw out the past relevant information compared to traditional RNN. The idea is to evaluate the first generated model and then decide which could be deployed in the EEBLab. Therefore, in this study, LSTM model performs well and has been selected to be exploited in the experiment, due to its effectiveness in terms of accuracy (LSTM 98.7%, GRU 97.5%) and root mean square error (RMSE) (LTSM 3.34, GRU 3.73) parameters. In fact, Apache Kafka has been used, in this case study, to consume the actual number of the occupancy, coming from the HOLSYS platform, to forecast the next 10 steps ahead, serving as a real time input for the MPC controller model.

### 3.4. MPC for Predective Control

The work presented in [29], presents the developed dynamic model for CO_2_-based MPC of a building’s VS. The model, describing this system, is a state-space model, which is based on the relationship between the input/output airflow rates and indoor CO_2_ concentrations. The MPC controller model was tuned to be deployed in a real case scenario, considering the real context of the EEBLab, in particular, the building space and occupancy number profile as well as the characteristics of the VS. Simulations have been conducted to the following highlights during the tuning of the MPC controller input parameters:

- The controller output provides a slow response to CO_2_ set point and occupancy changes if the prediction horizon is short (i.e., 2 ≤ P ≤ 5 steps ahead);
- The output of the controller acts faster on changes, which means that the controller’s prediction ability increases if the prediction horizon is long (i.e., P ≥ 10 steps ahead);
- A longer control horizon (i.e., M ≥ 10 steps ahead), the response of the controller output becomes too aggressive and therefore overshoots, which does not occur when using a small control horizon.

In fact, the control horizon M and the prediction horizon P inputs are the key design parameters of the MPC. They have a significant impact on its performance (i.e., settling/rise time and stability), especially in the presence of disturbances. 

**Figure 8:**
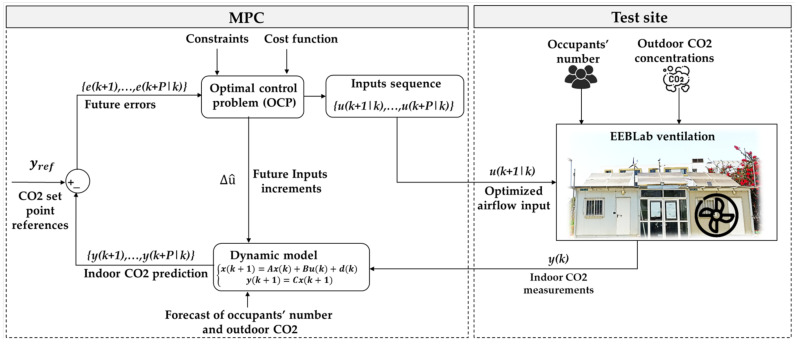
The general structure of the MPC framework for the EEBLab ventilation control system.

In this experimental study, as schematized in Figure 8, the optimal control problem (OCP) of the MPC is solved for every time interval (30 s) in which its optimal control output is calculated for the entire horizon P. The inputs to the OCP are the forecasted occupancy number, the outdoor CO_2_ concentration, and the previous measurement of indoor CO_2_ concentration (k−1), along with the system constraints (i.e., indoor CO_2_ set point and airflow limits).

The prediction and control horizons used in the MPC framework are respectively P = 10 (i.e., 300 s) and M = 5 (i.e., 150 s) steps ahead. For occupancy, the forecasted number is used to control the indoor CO_2_ dynamics, including the CO_2_ generated by the occupants over the horizon P. The forecast of the occupants’ number and measurements of the indoor/outdoor CO_2_ are forwarded to the OCP. The optimized control output (i.e., minimal required airflow) is fed back to the dynamic model to calculate the future predictions of indoor CO_2_ concentrations for the entire prediction horizon P. This calculation is repeated every time interval. At each time interval, the future occupancy and prediction of the indoor CO_2_ concentrations along with the constraints are updated and passed to the OCP to plan the next sequence of control inputs to be applied at that time. Only the first optimal input of the control sequence is implemented, and the remaining input values are discarded.

To solve the OCP, the following quadratic cost function is used, which reduces the future error $\hat{e}$ between the CO_2_ set point references $y_{ref}$ and predicted indoor CO_2_ concentration $\hat{y}$ through the prediction Horizon P. This is mainly achieved by applying the optimal control increment action $\Delta \hat{u}$ in which the minimum of airflow u is delivered and the indoor CO_2_ concentration y is maintained within comfort bounds. Q and R represent weighting matrices. The set point of indoor CO_2_ concentration is defined at 550 PPM, whereas the outdoor CO_2_ is kept at a constant value of 400 PPM.

$$\underset{\Delta u}{Minimize}J=\frac{1}{2}\sum_{K=0}^{k=P}\left[\left(\hat{y}-y_{ref}\right)Q{\left(\hat{y}-y_{ref}\right)}^{T}+\left(\Delta \hat{u}\right)R{\left(\Delta \hat{u}\right)}^{T}\right],$$

Subject to, y<550 PPM and $0<u<440\;m^{3}/h\;\left(\sim 0.12\;m^{3}/s\;\sim \;122.22\;L/s\;\sim \;259\;\mathrm{scfm}\right)$.

## 4. Real-Time Implementation

**Figure 9:**
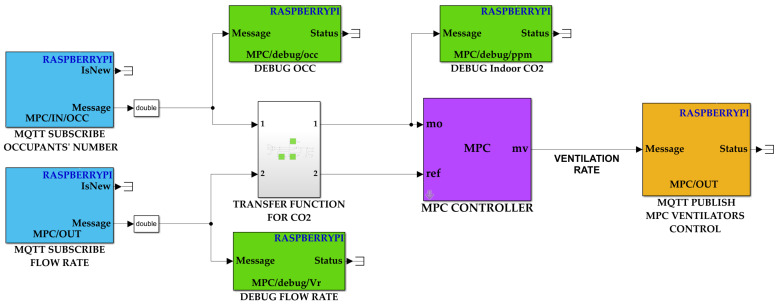
MATLAB/Simulink model for ventilation system’s control using MPC.

This section presents the implementation of the MPC framework for controlling the VS with the aim to improve both the indoor air quality and energy saving. An HIL experiment in which the MPC model controller is physically implemented in an RPi development board is conducted. Figure 9 shows the blocks that have been integrated to enable the communication between the MPC VS model, which is carried out with the MPC toolbox of MATLAB/Simulink environment and the HOLSYS platform.

To enable the use of MQTT in the MATLAB/Simulink model, the Raspberry Pi support package for MATLAB and Simulink have been installed using MATLAB Add-ons.

**Figure 10:**
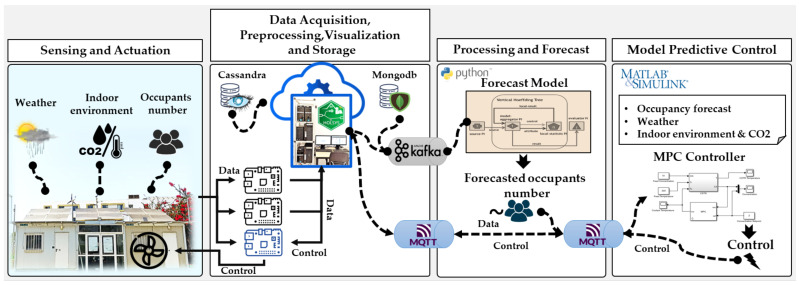
The experimental setup architecture of HIL implementation of the MPC for EEBLab ventilation system control enabled by the HOLSYS IoT platform.

Many blocks are available in the Simulink libraries under “Simulink Support Package for Raspberry Pi Hardware”. The two used blocks are “MQTT subscribe” and “MQTT publish”. The former subscribes to the topics “MPC/IN/OCC” and “MPC/IN/CO_2_” to get, respectively, the forecasted occupant’s number and CO_2_ measurements from the forecast model and the HOLSYS platform. The latter publishes the required flow rate into the “MPC/OUT” topic to control the ventilation speed. As shown in Figure 3b, the ventilation control node is wired to the inlet and outlet fans and controls them using PWM. In fact, the node receives the required flow rate from the platform through MQTT and transforms it to the corresponding PWM signal. The general architecture of the experimental setup from sensing till the control execution is illustrated in Figure 10.

The MPC controller, which is run from MATLAB/Simulink simulator, is emulated and embedded into the RPi as an independent hardware in the network.

However, Simulink is able to keep monitoring the simulation run time as well as the inputs and outputs of the MPC model. Using an MQTT publisher and subscriber tool, it is possible to inject test data into the model or monitor any variable from all over the experimental setup and its system entities.

## 5. Results and Discussions

In this section, the results obtained from the experimental setup of the different deployed systems are presented. In fact, after connecting everything together, the simulations have been run and data are collected during the experiment’s time. The experimentation started at 13:36 at the EEBLab test site. All the windows were closed and the only source of fresh air was the ventilation inlet. The HVAC was set to off. The weather station readings at the time were 23 °C for the ambient temperature and 56% for the relative humidity. The behavior of two employees at an office of 12 m^2^ was simulated. Other staff joined the team from time to time. While the occupants were performing moderate activities (e.g., using their personal computers and reading several articles while conversing), CO_2_ was changing its levels and increasing with more people inside the test site. At each new visit, the door was opened and closed in approximately 5 to 8 s. However, the influence of the door openings and the air exchanged during this time has not been taken into consideration. In fact, the objective behind the experimental setup is to show the usefulness of the proposed framework with all its components. It is a proof of concept of the intercommunication of different entities that form the entire concept. The idea is to integrate control strategies and modern technologies into a holistic framework for enabling real time monitoring and control of buildings’ systems.

First, advanced methods for forecasting indoor occupancy are implemented. Real occupancy data is sent to the server to be processed and exploited by the deployed forecasting model. It is implemented to read 10 instances and forecast 10 values ahead. The model gets new data every 1 min. It means that the model is able to forecast 10 min ahead. the forecasted occupancy data is sent to the MPC model to measure the flow rate needed to adjust optimal operation of the VS. The calculated accuracy and root mean square error (RMSE) parameters of the LSTM forecasting are respectively, 3.34% and 98.7%. Secondly, an MPC control strategy is integrated for VS’s control. All together, these systems have been inter-communicated via the deployed IoT platform. The simulation model of the MPC controller has been compiled via MATLAB/Simulink and embedded into the RPi 4 B+ installed into the test site. Afterwards, the installed sensors began to inject input data to the forecasting and MPC models and outputs (controls) were executed by the ventilators.

**Figure 11:**
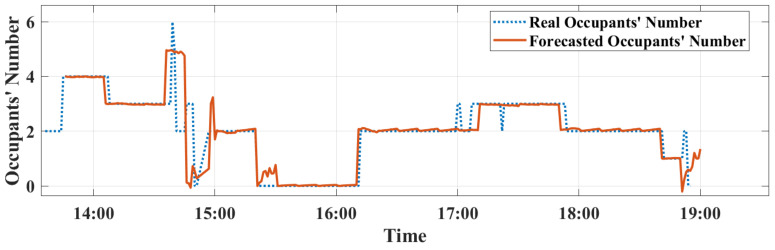
Occupancy forecasting results using LSTM.

**Figure 12:**
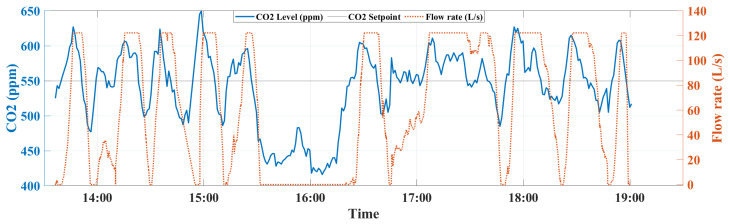
The MPC flow rate output together with the CO_2_ concentration variation.

**Figure 13:**
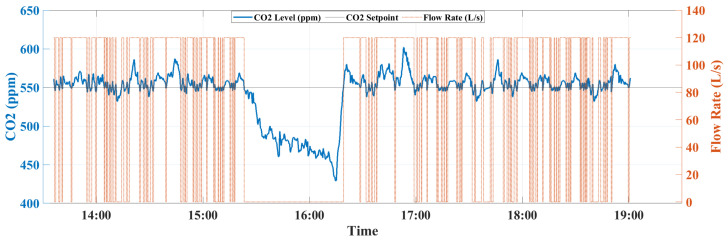
The ON/OFF flow rate output together with the CO_2_ concentration variation.

**Figure 14:**
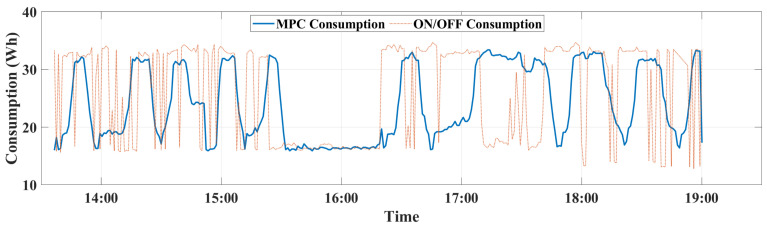
Energy consumption variation of the ventilation system during control for both ON/OFF and MPC controllers.

In order to assess the performance of the MPC against the ON/OFF, three metrics have been generated: (i) the regulation of indoor CO_2_ concentration, (ii) the ventilation flow rate evolution, and (iii) the instantaneous power consumption, which are calculated using the smart metering platform [26]. Experiments have been conducted using the above-mentioned set-up and the three metrics have been evaluated for the ON/OFF and MPC controllers during five hours and a half from 13:30 to 19:00. The occupants’ number together with the behavior of indoor CO_2_ concentration, ventilation flow rate, and power consumption of two controllers can be observed in Figure 11, Figure 12, Figure 13 and Figure 14. 

As can be seen from Figure 11, the forecasted occupants’ number seems to be close and to correlate well with the collected real occupants’ number. A minor difference is seen at the peak points of the real occupancy.

The ON/OFF approach has been chosen for comparison as it is the most used control approach in VSs. It is a simple control mechanism which triggers full On or full Off in case of CO_2_ variation from the fixed setpoint. The ON/OFF control was deployed using the above-mentioned approach the next day.

In terms of CO_2_ regulation, both controllers provide good performance in maintaining the CO_2_ concentration with faster settling/rise responses for the ON/OFF to achieve and maintain the desired level, which is fixed to 550 PPM setpoint. Unlike the ON/OFF, the MPC was able to provide a better transient response to refresh the air inside the EEBLab using the optimal ventilation rate, as can be observed from Figure 12 and Figure 13.

For energy consumption, the obtained results presented in Figure 14, showed that the MPC outperforms the ON/OFF and allowed higher performance in improving energy savings. This performance can be explained by the predictive mechanism of the MPC, which includes the optimized criterion $\Delta \hat{u}$ that predict the effective ventilation flow rate according to the indoor CO_2_ dynamics, including the CO_2_ generated by occupants.

Regarding the total energy consumption of the VS during this experiment time period, the MPC outperformed the ON/OFF control and allowed a significant energy reduction by 16.44%. It can be noticed from Figure 14, that the peak energy consumed by the ventilators is reached only a few times by the MPC control unlike the ON/OFF method. The total energy consumed by MPC control is 119.4 Wh while the ON/OFF consumed a total of 142.88 Wh.

## 6. Conclusions and Perspectives

In this work, an HIL based framework was introduced for standalone VSs using MPC control method. The objective was to assess the effectiveness of the proposed framework in terms of indoor air quality improvement and energy efficiency in real-setting scenario. In fact, a Simulink based HIL model was proposed and implemented in the EEBLab to assess the effectiveness of MPC control. Contextual data are collected using the HOLSYS IoT platform and LSTM machine learning models have been integrated for real time occupants’ number forecasting. Resulting forecast data have been exploited by the MPC for optimal regulation of the ventilation flow rate. The performance of the MPC over the HIL framework has been assessed and compared to the ON/OFF strategy. Experimental results showed that both controllers provide acceptable performance in regulating the indoor CO_2_ concentration. However, the MPC allowed significant energy reduction by approximately 16% compared to ON/OFF.

As a perspective of this work, the framework will be applied and experimented for the HVAC system’s control using MPC. This latter has already been validated by simulations [66]. Furthermore, additional experiments will be conducted to shed more light on the integration of IoT and machine learning algorithms for setting up context-driven control approaches of different building services, including lighting, shading, and HVAC systems. Additionally, the perspective includes integrating the proposed framework for developing other buildings services, such as renewable energy production forecasting and predictive control of power systems [24].

## Data Availability

Not applicable.
